# Actinobacteria Derived from Algerian Ecosystems as a Prominent Source of Antimicrobial Molecules

**DOI:** 10.3390/antibiotics8040172

**Published:** 2019-10-01

**Authors:** Ibtissem Djinni, Andrea Defant, Mouloud Kecha, Ines Mancini

**Affiliations:** 1 Laboratoire de Microbiologie Appliquée, Faculté des Sciences de la Nature et de la Vie, Université de Bejaia, Targa Ouzemmour 06000, Algeria; kmkmsetif@yahoo.fr; 2 Bioorganic Chemistry Laboratory, Department of Physics, University of Trento, 38123 Trento, Italy; defant.andrea@unitn.it

**Keywords:** actinobacteria, antimicrobial, antibiotic, antifungal, secondary metabolites, chemodiversity, rare actinobacteria, Saharan ecosystem

## Abstract

Actinobacteria, in particular “rare actinobacteria” isolated from extreme ecosystems, remain the most inexhaustible source of novel antimicrobials, offering a chance to discover new bioactive metabolites. This is the first overview on actinobacteria isolated in Algeria since 2002 to date with the aim to present their potential in producing bioactive secondary metabolites. Twenty-nine new species and one novel genus have been isolated, mainly from the Saharan soil and palm groves, where 37.93% of the most abundant genera belong to *Saccharothrix* and *Actinopolyspora*. Several of these strains were found to produce antibiotics and antifungal metabolites, including 17 new molecules among the 50 structures reported, and some of these antibacterial metabolites have shown interesting antitumor activities. A series of approaches used to enhance the production of bioactive compounds is also presented as the manipulation of culture media by both classical methods and modeling designs through statistical strategies and the associations with diverse organisms and strains. Focusing on the Algerian natural sources of antimicrobial metabolites, this work is a representative example of the potential of a closely combined study on biology and chemistry of natural products.

## 1. Introduction

By receiving the Nobel Prize for the discovery of penicillin in 1945, Alexander Fleming informed the scientific community that the misuse of antibiotics would lead, in the near future, to the emergence of microbial pathogens resistant to these substances. Fleming’s prediction was true as we are confronted in recent decades with the emergence of multidrug-resistant bacteria that threaten global health. [1]. The inappropriate use of antibiotics has created a selective pressure that drives the emergence and spread of multidrug-resistant pathogens, like those of the ESKAPE group (*Enterococcus faecium*, *Staphylococcus aureus*, *Klebsiella pneumoniae*, *Acinetobacter baumannii*, *Pseudomonas aeruginosa*, and *Enterobacter* spp.), to all antibiotics currently used in therapy. Bacteria have developed diverse resistance mechanisms to avoid antimicrobial agents action, classified as follows: (i) antimicrobial molecule modification, (ii) decrease in permeability or extrusion of the antimicrobial compound through the overexpression of efflux pumps, (iii) changes and/or bypass of target sites by mutations in their encoding genes (either by protecting or modifying the target site), and (iv) resistance due to global cell adaptions. Moreover, new resistance mechanisms are constantly being described, for example, for colistin, which is the last-resort antibiotic for multidrug-resistant bacteria infections [2]. Despite the alarming situation, the number of new antibiotics placed on the market has been in decline in recent years. At the present, the limited choice of antibiotic classes for treating multidrug-resistant bacteria encourages scientists to search for unknown molecules displaying new mechanisms of action.

Natural microbial sources are considered the most important source of bioactive metabolites that are promising for new therapeutic drugs [3,4,5]. The *Actinobacteria* phylum represents one of the largest taxonomic groups in the *Bacteria* domain. It includes mycelium-forming or not, Gram-positive bacteria, with a high G + C content reaching 70% for some species of the *Streptomyces* genus. As might be expected from a large phylum, representatives of *Actinobacteria* are found in a wide range of ecological niches, including aquatic ones such as marine and oceanic sediments, seawater, freshwater ecosystems, and marine invertebrates. In terrestrial environments, different lifestyles are found in actinobacteria like plant commensals, nitrogen-fixing symbionts, as well as animal and plant pathogens. Thus, they constitute a significant proportion of the telluric microflora [6]. 

*Streptomyces* genus is known for its complex development cycle which has been extensively studied, indicating that secondary metabolites are synthesized as a defence against antagonistic microorganisms and also ensuring a major role in the cycling of organic matter in the soil and sediments ecosystem. Details on this topic have been efficiently reviewed [7,8]. The filamentous actinobacteria belonging to the *Actinobacteriaceae* family have the potential to produce chemically diverse and relevant metabolites counting known antibiotic, antifungal, antitumor, and anti-inflammatory agents, along with plant-growth-promoting substances and regulators. These substances find different applications including medicine, biotechnology, and agriculture [9] in addition to the industrially relevant enzymes (e.g., cellulases, chitinases, and xylanases) responsible for the production of biofuels and biochemicals [10,11]. The actinobacteria genome is rich in biosynthetic gene clusters (a group of two or more genes that together encode a biosynthetic pathway) coding for known and/or novel metabolites with potential to discover new therapeutic agents [12,13]. In particular, actinobacteria synthesize a large number of bioactive metabolites of which antibiotics cover the major proportion. For almost a century, actinobacteria have contributed significantly to the development of the antibiotic arsenal required for human health, so they are responsible for the production of more than 70% of relevant anti-infective natural products. Antimicrobial agents have been the first isolated natural compounds, starting from actinomycin from *Streptomyces antibioticus* in 1940, followed by a significant number of antibiotics discovered in the so-called “golden age” corresponding to the period 1940s–1960s, when the production of about half of all known antibiotics is due to *Streptomyces* [14]. 

Besides antibacterials, the interest was on the search for new and more effective antifungal agents, particularly against opportunistic molds and fungal infections caused by *Aspergillus* and *Candida albicans*, the latter responsible of nosocomial infections. Furthermore, while the frequency of fungal infections is increasing alarmingly, current antifungal therapy has a limited number of drugs due to their side effects and toxicity. In addition to antibiotics and antifungals, actinobacteria are known to produce a wide range of secondary metabolites with a broad range of bioactivities including antitumor, antioxidant, and herbicide- and plant-promoting agents. 

Although actinobacteria are known for their rich metabolism, it has become always more difficult to find novel bioactive substances due to the frequent rediscovery of already known compounds. One of the main strategies in the search for new sources of bioactive compounds is the isolation of rare actinobacteria (non-Streptomycete actinobacteria) from underexplored and uncommon habitats [15]. The potential of this approach has been extensively reported [16,17] since the first isolation in 1964 of a thermophilic actinobacterial strain from an Italian soil sample, which led to discovery of the antibiotic thermorubin [18]. Moreover, conditions such as temperature, light radiation, and salt concentration of arid and semiarid ecosystems proved capable in affecting the metabolite profile of the extremophilic actinobacteria [19].

It must be remembered the highly complex structures of many bioactive metabolites from actinobacteria and the rich presence of stereogenic centres with defined configurations due to the enantioselective synthesis occurring in nature. Therefore, the employment of suitable bacteria strains still remains the method of choice for their production. It is a more advangeous method than the organic synthesis to obtain enantiomerically pure forms of bioactive molecules and in large-scale access for therapeutic applications.

To this purpose, a wide range of methods have been used for the selective isolation of actinobacteria and have applied combined physical and chemical approaches, such as thermic treatments [20], or the addition of chemicals (calcium carbonate and chitin, calcium chloride, phenol [21,22,23,24], and sodium dodecyl sulfate). The incorporation of antibacterial (e.g., nalidixic acid and kanamycin) and antifungal agents (e.g., nystatin and amphotericin B) to the culture media is also an effective approach for the selective isolation of these bacteria.

Another aspect is related to the generally low production of secondary metabolites. To overcome this restriction, a particular attention was focused on the employment of both conventional and new methodologies able to enhance their production. It is widely accepted that culture parameters significantly affect the performance of microbial metabolism [25]; therefore, an optimization of culture media and physical chemical conditions of the fermentation process usually comes before any production on a large scale. Different upscaling techniques are also used by industries for yield maximization during both the fermentation process and the extraction steps [26].

In detail, one factor at time (OFAT) approach has been widely applied in the past for any upscaling production due to its simplicity [27], although it was reduced mainly during the initial steps of medium formulation due to its drawbacks (time-consuming process, expensive, needs many experiments, and parameter interactions not taken into account). It has been replaced by statistical methodologies such as design of experiments procedure (DOE), which is more effective, quick, and accurate, requiring less experiments; and estimating the effects of several culture parameters simultaneously [28,29]. Through statistical strategies of culture conditions, the Plackett–Burman design (PBD) and the Tagushi design (TD) have been successfully applied as modeling methodologies to evaluate the culture parameters affecting the metabolite production. The most widely used tools for the optimization step are central composite (CCD) and Box and Behnken designs (BB) in response surface methodology (RSM) [30].

Mixed cultivation of actinobacteria/actinobacteria, actinobacteria/bacteria, or actinobacteria/ fungi allows to activate cryptic biosynthetic pathways. Co-cultivation at a laboratory scale can reproduce the original and natural conditions of microorganisms, creating an antagonism and competitive environment able to stimulate and enhance the production of new secondary metabolites. The effectiveness of this strategy is well represented by the production of new bioactive molecules derived from the dual culture of desert-derived isolated *Streptomyces leeuwenhoekii* strains with a marine-derived fungi *Aspergillus fumigatus* [31]. 

An additional attractive source of novel bioactive compounds is given by the endophytic actinobacteria acting as biocontrol agents of plant disease, responsible for plant-promoting growth via the production of diverse substances [32], in particular, screening of native plants from the Algerian Sahara to isolate endophytic actinobcateria for the biocontrol of *Rhizoctonia solani* damping-off and the improvement of tomato growth [33,34], which is in line with the microbial symbiotic association with insects and which led to the discovery of a number of new and diverse chemical structures [35]. 

Moreover, genome analysis of actinobacteria turned out to be a promising approach for the study of the metabolic potential and identification of novel biosynthetic gene clusters. Indeed, genomic sequencing has revealed the presence of silent genes and cryptic biosynthetic pathways encoding for secondary metabolites not expressed under conventional culture conditions [36]. The significant success of the genomic tools led to the exponential increase of the number of available actinobacteria genome sequences reported in suitable databases. Furthermore, ribosome engineering has been successfully applied to increase the production of bioactive secondary metabolites, in particular, to obtain antibiotics from *Streptomyces* [37]. 

In the present review, we highlight the diversity of actinobacteria coming from ecosystems in Algeria and the chemical structures of the corresponding secondary metabolites associated to their biological activities. Moreover, the attention is focused on the novel species and genera studied and on new bioactive compounds reported since 2002. Additionally, we discuss the biosynthesis improvement of these metabolites through the application of classical and advanced methodologies. Data reported in this paper were obtained from PubMed and SciFinder (Chemical Abstract online) databases. To the best of our knowlege, this is the first overview on the biology and chemistry of actinobacteria from Algerian ecosystems reported so far.

## 2. Algerian Sampling Sites Providing Culturable Actinobacteria 

**Figure 1:**
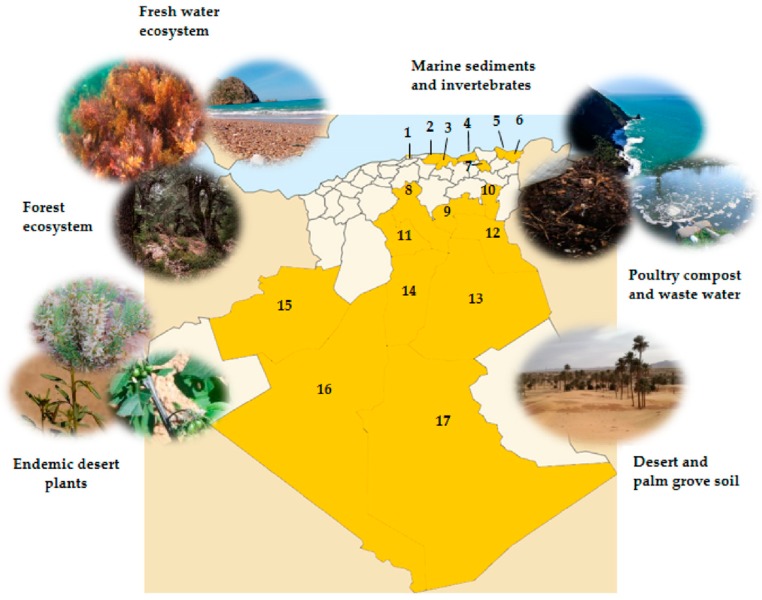
Distribution of the most explored *Actinobacteria* sampling sites in Algeria. **1**: Algiers, **2**: Tizi Ouzou, **3**: Bejaia, **4**: Jijel, **5**: Annaba, **6**: El Taref, **7**: Constantine, **8**: Djelfa, **9**: Biskra, **10**: Khenchela, **11**: Laghouat, **12**: El Oued, **13**: Ouargla, **14**: Ghardaia, **15**: Bechar, **16**: Adrar, and **17**: Tamarasset. Details are in Table 1.

With an area of more than 2 million square kilometers, Algeria has an impressive climatic diversity ranging from snow-capped mountains in the northern regions overlooking the Mediterranean Sea to the world’s hottest Saharan desert. This affects a great biodiversity, rich and diversified in actinobacteria, to which corresponds a wide chemodiversity of metabolites. A number of actinobacteria has been isolated from different ecosystems including Saharan plants [34,38,39], caves [40], waste water [41,42], river sediments [43], hypersaline areas [44,45,46,47,48,49,50,51], Saharan desert soil [22,52], and derived algae [53]. The most studied sampling sites for the isolation of actinobacteria are listed in Figure 1.

**Figure 2:**
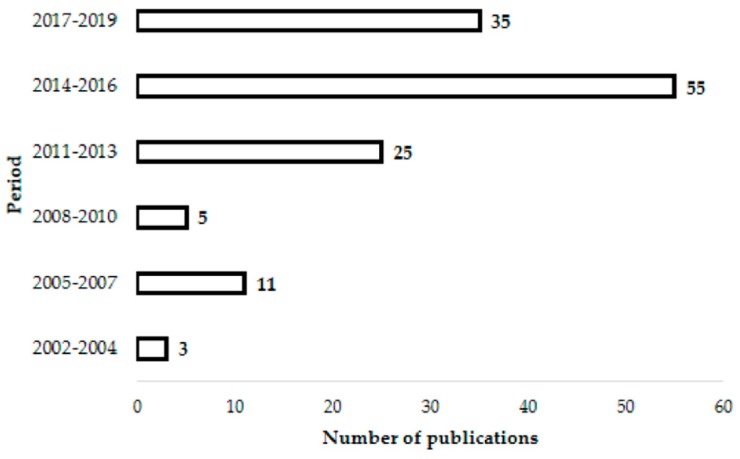
Variation in the number of publications dealing with the isolation and metabolic profile investigation of actinobacteria in Algeria based on data since 2002 to the present.

Under the research topic of *Actinobacteria* as a resource of novel potential therapeutic agents, a total of 134 articles were published starting from 2002 (Figure 2). They cover different aspects, including the isolation and diversity of bacteria along with the investigation on secondary metabolite production targeting a broad spectrum of bioalogical activities (mainly antimicrobial but also cytotoxic and plant promoting agents) and with a look at potential biotechnological applications [54].

## 3. Biodiversity of Rare and Novel Genera and Species of Actinobateria

**Figure 3:**
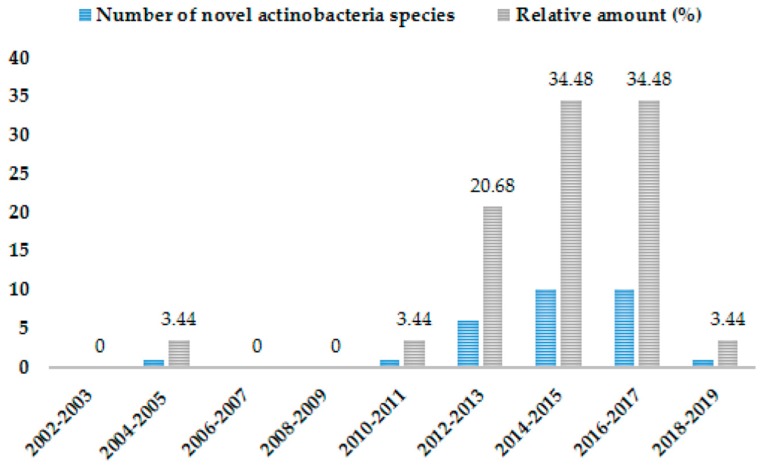
Variation in number of culturable novel actinobacteria species collected from Algerian sources since 2002.

The interest in actinobacteria biodiversity present in Algerian ecosystems started more than two decades ago [55,56,57,58]. From a historical point of view, the research activities carried out in 1974 by the group of Bounaga, director of the arid zone research center (CRZA) in Algeria, focused on biological control methods including the use of actinobacteria against the appearance of Bayoud’s disease caused by *Fusarium*, a serious date palm disease that threatens all date-producing countries. The Sabaou team started within CRZA, and it was only in 2000s that the activity in this field improved due to the official creation of university research laboratories. The studies by Sabaou et al. have shown the great potential of Algerian habitats for novel actinobacteria species, indicating the richness of their metabolism for the production of bioactive compounds [22]. Thereafter, a large number of new species have been discovered, mainly from the Saharan ecosystem, which represents 85% of the total area of the country (Figure 3). The number of novel species and rare actinobacteria isolates reported from the Algerian ecosystems [24,44,45,46,47,48,49,50,59,60,61,62,63,64,65,66,67,68,69,70,71,72,73,74,75,76,77,78,79,80] has increased in the last years (Table 1), with the discovery of six new species between 2012 and 2013, to reach a peak of 20 new species and one genus between 2014 and 2017 and a total of 10 species within 2 years in the periods of 2014–2015 and 2016–2017 (Figure 3).

**Figure 4:**
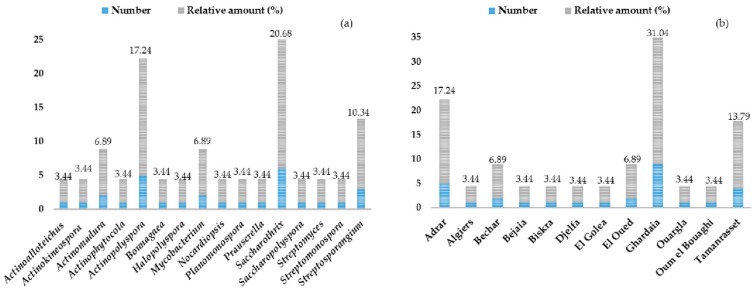
Proportion of novel species, genera, and rare actinobacteria strains from different Algerian ecosystems reported since 2002 (**a**), according to the places of collection (**b**). Details are in Figure 1.

Figure 4a illustrates the proportion of novel species, genera, and rare actinobacteria described since 2002 from different Algerian ecosystems. More than 29 novel species and the new genus *Bounagaea* have been discovered, as was the new family *Mzabimycetaceae* proposed by Saker et al. [62]. The same authors published *Mzabimyces algeriensis* as a novel strain isolated from a palm grove soil sample of the Mzab region-Ghardaia (south of Algeria). It must be specified that the strain was recently reclassified regarding the genus *Halopolyspora* and identified as *Halopolyspora algeriensis* comb. nov. by comparison of phenotypic, chemotaxonomic, and phylogenetic data and DNA–DNA hybridization [63]. Among 29 new species and 16 genera belonging to the order *Actinomycetales*, *Saccharothrix* is the most recovered and abundant genus, accounting for 20.68% of the total novel actinobacteria species from Southern Algeria regions, followed by the *Actinopolyspora* genus with a proportion of 17.24%. In summary, both amount and diversity of rare actinobacteria genera deriving from the Saharan desert evidence the wealth of this particular ecosystem.

**Figure 5:**
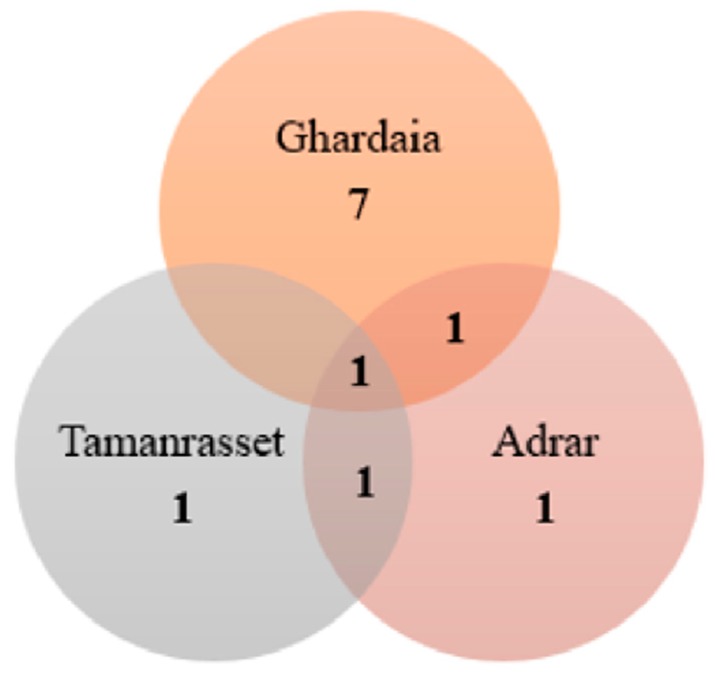
Venn diagram showing the numbers of shared and unique rare actinobacterial genera among the three most dominant Saharan soil samples sites.

The sampling sites regarded for the isolation and investigation of rare actinobacteria have been essentially focused on the Saharan soil and palm groves. The Adrar, Ghardaia, and Tamanrasset regions are together associated with the discovery of 18 new species: *Saccharothrix algeriensis* sp. nov., *Saccharothrix saharensis* sp. nov., *Nocardiopsis algeriensis* sp. nov., *Actinomadura adrarensis* sp. nov., *Streptosporangium algeriense* sp. nov., *Saccharopolyspora ghardaiensis* sp. nov., *Actinopolyspora mzabensis* sp. nov., *Halopolyspora algeriensis* comb. nov., *Actinokineospora mzabensis* sp. nov., *Saccharothrix isguenensis* sp. nov., *Actinophytocola algeriensis* sp. nov., *Streptosporangium saharense* sp. nov., *Saccharothrix ghardaiensis* sp. nov., *Saccharothrix hoggarensis* sp. nov., *Saccharothrix tamanrassetensis* sp. nov., *Actinomadura algeriensis* sp. nov., and *Actinoalloteichus hoggarensis* sp. nov., belonging to 11 different genera (Figure 4b). A Venn diagram assembling shared and unique genera obtained from each of the three studied regions (Adrar, Ghardaia, and Tamanrasset) is presented in Figure 5. Just one genus, *Saccharothrix*, shared across the Adrar, Ghardaia, and Tamanrasset sites, represents the most abundant recovered genus from the arid soil. The Adrar and Tamanrasset locations shared only the *Actinomadura* genus, and the Adrar and Ghardaia sites showed *Streptosporangium* as the common genus. Thus, so far, the Saharan desert is associated with 28 new species of actinobacteria that are related to soil and palm groves.

## 4. Secondary Metabolites Derived from Algerian Actinobacteria 

**Figure 6:**
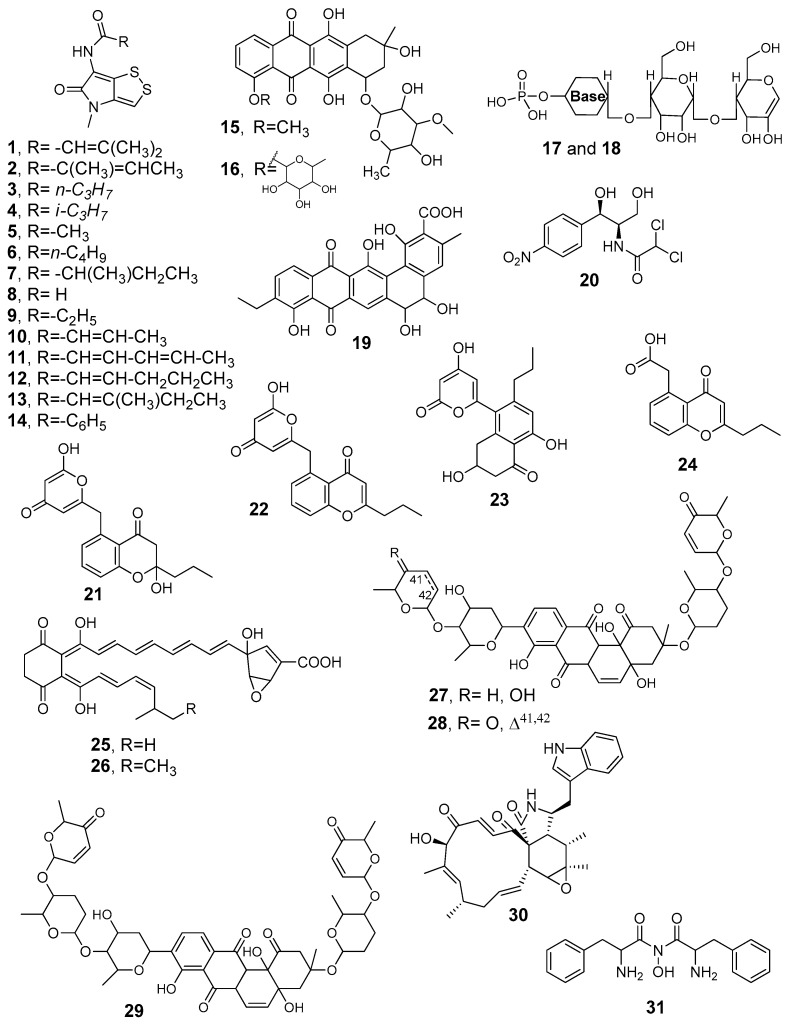
Molecular structures of the bioactive metabolites **1**–**31** isolated from Algerian actinobacteria: The stereochemical details are according the cited references.

**Figure 7:**
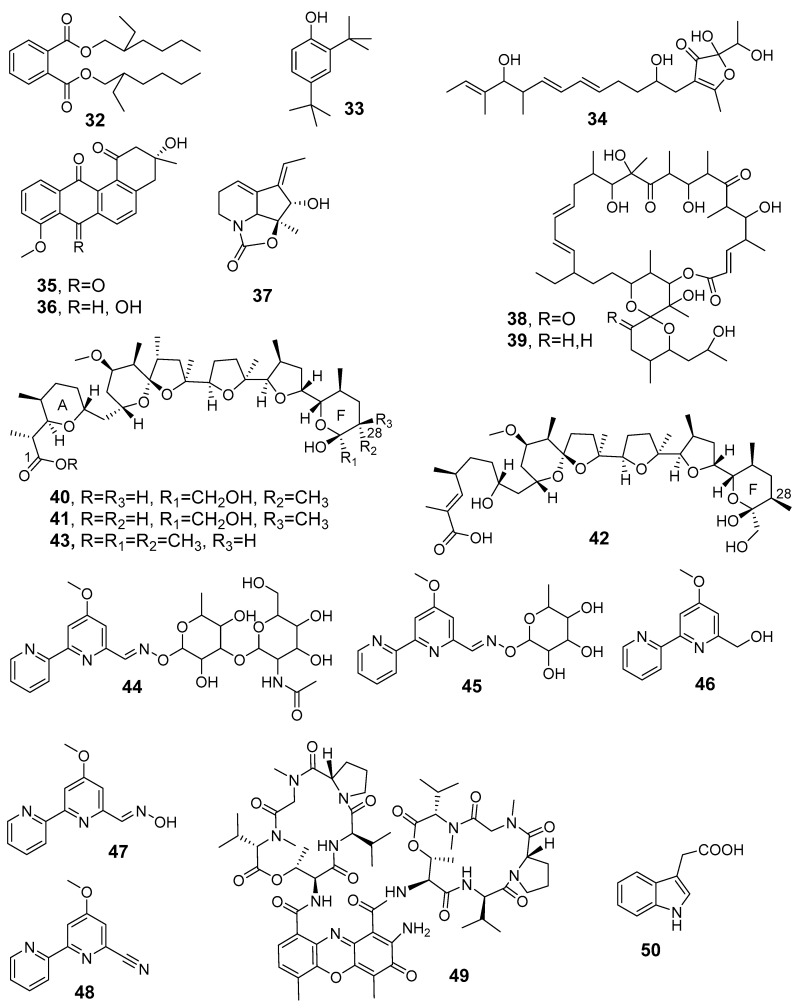
Molecular structures of the bioactive metabolites **32**–**50** isolated from Algerian actinobacteria. The stereochemical details are according the cited references.

Bioactive natural products are reported to be classified according to their biological activities, focusing on isolation and elucidation of their chemical structures (**1**–**50** in Figure 6 and Figure 7) and on their production by both classical changes of culture conditions and new statistical approaches, when present in the original works. Table 2 lists the metabolites, summarizing their data on sources, producer strains, and bioactivies.

### 4.1. Antimicrobials

Lamari et al. [23] isolated the new dithiopyrrolone metabolites, namely 3-methyl-2-butenoylpyrrothine (**1**), tigloylpyrrothine (**2**), and *n*-butyropyrrothine (**3**), along with the known iso-butyropyrrothine (**4**) and thiolutine (**5**) from a Saharan palm grove soil strain *Saccharothrix* sp. SA233 (Figure 6). These products were isolated after a workup including centrifugation of the fermentation broth, extraction with dichlormethane and purification by preparative thin layer chromatography (TLC) on silica gel, followed by reversed phase HPLC. The new metabolites (**1**)–(**3**) displayed high antibacterial activity against *Bacillus coagulans*, *Bacillus subtilis*, and *Micrococcus luteus*, specifically with minimum inhibitory concentration (MIC) values of <0.2, 0.5, and 1 µg/mL (corresponding to <0.7, 1.8, and 3.9 µM), respectively, against *Bacillus coagulans*. The three compounds exhibited also a higher activity against *Saccharomyces cerevisiae* and *Mucor ramanniamus* compared to the phytopathogenic fungi *Fusarium oxysporum* f. sp. *albedinis*, *F. o.* f. sp. *Lini*, and *F. culmorum*. The structural characterization of the metabolites was reported by the same authors [81]. Later, from the fermentation broth of *Saccharothrix algeriensis* NRRL B-24137 (fully sequenced strain) isolated from a Saharan soil sample collected in 1992 at a palm grove in Adrar (southwest of Algeria), Merrouche et al. [82,83] isolated the new dithiolopyrrolone derivatives valerylpyrrothine (**6**), isovalerylpyrrothine (**7**), and formylpyrrothine (**8**) by addition of valeric acid to the culture medium and the known aureothricin (**9**), exhibiting a moderate bioactivity against some filamentous fungi and yeasts such as *Mucor ramanniamus*, *Penicillium expansum*, and *Aspergillus carbonarius*. Moreover, the new crotonyl-pyrrothine (**10**), sorbyl-pyrrothine (**11**), 2-hexonyl-pyrrothine (**12**), and 2-methyl-3-pentenyl-pyrrothine (**13**) were obtained by addition of sorbic acid to the culture medium. Compound **11** resulted in the most active in the series against Gram-positive bacteria. All metabolites showed a moderate inhibition of the tested fungi and yeast, except compound **13**, which gave a higher activity against *Aspergillus carbonarius* and *Candida albicans* with MICs evaluated at 2 µg/mL (MIC = 7 µM) for both germs [83]. Very recently, the same authors have reported on the new benzoyl-pyrrothine dithiolopyrrolone (**14**), obtained after the supplementation of cinnamic acid into the culture medium of *S. algeriensis* NRRL B-24137. In the antibacterial evaluation, *Listeria monocytogenes* resulted in the most sensitive to the treatment with this pure metabolite (MIC = 4 µg/mL corresponding to 13.8 µM) [84].

Zitouni et al. [85] purified the new anthracycline antibiotic mutactimycin PR (**15**) and the already reported mutactimycin C (**16**) from the *Saccharothrix* sp. SA 103 strain collected in the Tamanrasset, an arid region of Southern Algeria. The culture broth was extracted using *n*-butanol and the residue subjected to reversed phase HPLC under isocratic conditions to give the pure metabolites. The compounds displayed moderate activity against some Gram-positive bacteria and fungi, especially *Bacillus subtilis*, *Saccharomyces cerevisiae*, and *Mucor ramannianus*. Some of the same authors isolated also 54 *Nocardiopsis* and 32 *Saccharothrix* strains from Algerian Saharan soils, identified by morphological and chemotaxonomic features. These strains showed antibacterial (against *B. subtillis* and *M. lutesus*) and antifungal activity against *M. ramannianus* and *S. cerevisiae* and also against mycotoxinogenic fungi *Fusarium graminearum*, *F. solani*, *F. culmorum*, *Aspergillus carbonarius*, *A. ochraceus*, and *penicillium citrinum*. A medium containing ammonium sulfate, starch, and yeast extract produced nucleotidic and nucleosidic molecules named ZA01 (**17**) and ZA02 (**18**), which were HPLC purified and of which their structures were partially characterized by electrospray–mass spectrometry (ESI-MS) analysis including tandem fragmentation experiments [86]. 

The phylogenetic analysis on a strain collected from the Saharan soil in a southwest location of Algeria identified the *Streptomsporangium* Sg10 strain as a potential new genomic species. It produced compounds active against Gram-positive bacteria and fungi. Only a partial structural characterization was described, able to identify a generic glycosylated aromatic nature of these metabolites [87]. The same authors reported also on the actinomycete strain *Streptomsporangium* sp. Sg3 collected from Adrar (southwest of Algeria), which produced three pigments, called R1, R2, and R3, that display no activity against fungi and Gram-negative bacteria, with the highest inhibition against Gram-positive bacteria shown by R2. UV-visible, IR, and NMR spectrtoscopic analyses allowed to give a partial elucidation, supporting a quinone-anthracycline aromatic structure for these pigments [88]. Later, the authors established the structure of R2 by extensive NMR analysis and high-resolution mass spectrometry as the new angucyclinone (**19**), related to tetracyclines and anthracyclines. In detail, the optically active molecule was defined in its planar structure and no streochemical assignments have been reported. MIC values evaluated by the conventional agar dilution method showed potent activities against *Micrococcus luteus* ATCC 9314 and *Bacillus subtilis* ATCC 6633 (MICs = 0.5 and 1 µg/mL, corresponding to 1 and 2 µM, respectively) [89].

The novel isolate recovered from a desert soil sample collected in Beni-Abbes (southwest Algeria) and named *Nonomuraea* sp. NM94 was studied under liquid fermentation condition. It produced five bioactive compounds, which were HPLC purified and partially characterized by IR, ^1^HNMR, and ESI-MS investigation. It was only possible to define the same chemical class for all compounds, containing an aromatic unit substituted by aliphatic chains. In detail, one of the metabolites showed a molecular mass of 340 Da, as established by ESI-MS spectra recorded in negative ion mode. The crude dichloromethane extract of the strain was evaluated by a paper disc method, resulting in active against some Gram-positive bacteria, yeast, and fungi [90].

*Saccharothrix* sp. PAL54A strain isolated from a Saharan soil in Ghardaïa produced the known chloramphenicol (**20**); therefore, it is the first production of this antibiotic by a *Saccharothrix* species. [91]. 

Actinobacteria of marine origin and, in particular, marine endophytic actinobacteria are also promising sources of new classes of antimicrobial compounds. Mutualistic or parasitic interactions of actinobacteria with marine macroorganisms and invertebrates have been proven to affect the production of novel metabolites. One of the most representative examples is the production of the new polyketide **21** along with phaeochromicins B (**22**), C (**23**), and E (**24**). The metabolites were isolated from a solid-state fermentation of *Streptomyces* sp. WR1L1S8 obtained from a marine brown algae *Fucus* sp. The structure of **21** was established regarding its 2-hydroxy-γ-pyrone tautomeric form by both NMR study on the products from deuterium incorporation using CD_3_OD solvent and the comparison of experiments with density functional theory (DFT)-calculated IR spectra. The Cotton effect observed by circular dichroic analysis is in favor of the enantiomeric purity of the natural product, denying the idea to be a product by water addition during the workup. However, the absolute configuration of the molecule remains undetermined. The new metabolite represents the lacking member in the series of phaeochromycins A–E, which are the first polyketides bearing the *n*-propyl chain. Compound **21** exhibited a selective bacteriostatic activity against methicillin-resistant *Staphylococcus aureus* (MRSA) (MIC = 6 μΜ) [53]. Culture conditions on antibacterial activity and mycelial growth were later evaluated, changing the parameters able to affect the production of metabolites **21**–**24**. The optimal conditions to increase the yield of the new anti-MRSA compound **21** were established using the OFAT approach on the culture of the strain *Streptomyces sundarbansensis* WR1L1S8 (on the starch casein agar medium in freshwater or 50% seawater at pH 7 or 9 at 28 °C using agar-state fermentation). In this study, the analysis carried out by HPLC equipped with a diode array detector evaporative light scattering detection (DAD-ELSD) or online coupled to an ESI-MS apparatus emerges as an efficient method to evaluate the chemical profile of the metabolites present in the crude extracts derived by different culture comditions. Compound **21** resulted in being also the most abundant by culturing the strains on starch casein agar medium in freshwater or 50% seawater at pH 7 or 9 using agar-state fermentation method [92].

The novel *Saccharothrix* SA198 strain from a Saharan soil sample (collected at Tamanrasset in southern Algeria at a 1370-m altitude) provided the new antibiotics A4 (**25**) and A5 (**26**). Their production was evaluated by changing culture media and pH values, and they were HPLC purified starting from the crude dichloromethane extract. The planar structures of **25** and **26** were established by MS data and 2D-NMR analysis. Pure metabolites displayed moderate activities against Gram-positive and -negative bacteria and potent effects against phytopathogenic and toxinogenic fungi: *Mucor ramannianus* (MICs: 5 μg/mL for **25** and 1 μg/mL for **26**), *Aspergillus carbonarius* (MICs: 10 μg/mL for **25** and 2 μg/mL for **26**), and *Penicillium expansum* (MICs: 2 μg/mL for each **25** and **26**) [93].

The anthracycline saquayamycin A (**27**) and C (**28**), known for their antibacterial and anticancer activities, were obtained from a culture broth of a novel *Streptomyces* spp. PAL114 strain collected in Ghardaïa. MIC values of pure compounds were evaluated using conventional agar dilution method on a series of microorganisms, observing moderate activities, with the highest effects against *Bacillus subtilis* ATCC 6633 and *Candida albicans* M3 [94]. 

Another metabolite belonging to the family of anthracyclines, the aquayamycin-like vineomycin A1 (**29**), was purified from the same strain: *Streptomyces* sp. PAL114. The strain produced also the cytochalasin derivative chaetoglobosin A (**30**). It is remarkable that chaetoglobosin A is known to be produced only by fungi and that this is the first report in prokaryotes. Both metabolites exhibited moderate effects against *B. subtilis* and *Candida albicans* and on filametous fungi [95]. 

The novel hydroxamic acid (**31**) was purified from the culture broth of *Streptomyces* WAB9, a strain isolated from the Saharan soil collected in Bechar region. The pure compound was obtained by HPLC purification of the *n*-butanol extract from the culture filtrate, and its planar structure was established by ESI-MS spectra recorded in negative ion mode and extensive NMR investigation. It exhibited antibacterial activitiy towards a range of multidrug-resistant microorganisms, in particular, *Pseudomonas aeruginosa* IPA1 (10 µg/mL = 30 µM) and *E. coli* E52 (20 µg/mL = 60 µM) [96]. 

Driche et al. [97] reported the isolation of di-(2-ethylhexyl) phthalate (**32**) from the novel strain *Streptomyces* sp. G60 obtained from a Ghardaia soil sample by a workup including the use of a series of organic solvents (*n*-hexane, dichloromethane, and n-butanol and ethyl acetate). There is doubt that the compound is an actual metabolite, although the molecules have been also reported isolated from other natural sources, as cited by authors themselves. In fact, it is known that di-(2-ethylhexyl) phthalate (DEHP) is the most common member of the phthalates class used as a plasticizer. Moreover, the solvent power able to extract this plasticizer from polymeric bags indicated *n*-hexane, methanol, chloroform, and ethyl acetate, in increasing order [98]. Compound **32** was tested for its activity against different strains of *Staphylococcus aureus* and MRSA, obtaining strong effects [97].

Belghit et al. [99] isolated 2,4-di-tert-butylphenol (**33**) from a culture of *Streptomyces mutabilis* strain from a Saharan soil collected in Metlili (Ghardaïa region). The known compound was active against pathogenic fungi exhibiting a MIC value of 5 µg/mL against *C. albicans* M3. 

From the broth culture of the novel strain *Streptomyces* sp. AT37 obtained from Adrar Saharan soil (southwest Algeria) the furanone derivative **34** was detected by bioautography of the crude extract, purified by reversed phase HPLC, and identified as the known as antibiotic E-975. The compound exhibited a moderate activity against multidrug-resistant *S. aureus* and inhibited the biofilm formation, which were reduced by 50% at a concentration of 10–15 μg/mL [100].

*Nocardiopsis* species are known to be present in Saharan soils, characterized by saline and hypersaline properties. The new halotolerant *Nocardiopsis* sp. HR-4 strain, collected from the salt-lake soil named Sebkha of Ain, provided two angucyclinone aromatic polyketides. In particular, the stereochemistry of the known (−)-7-deoxy-8-*O*-methyltetrangomycin (**35**) was assigned by comparison with the polarimetric value obtained for the same molecule by stereoselective total synthesis [101]. Compound **36**, corresponding to the reduced form of one carbonyl group in the quinone unit, had been already isolated from an Indonesian *Streptomyces* spp. and reported without the stereochemical assignment at this centre [102]. These metabolites exhibited good antibacterial activities only against Gram-positive bacteria [103].

Recently, Djinni et al. [43] purified (+)-streptazolin (**37**), produced as a major compound in an appreciable amount from *Streptomyces thermoviolaceus* SRC3, a fresh-water sediment-derived strain. The structural characterization of streptazolin, including its absolute configuration previously defined by X-ray crystallographic analysis on a derivative, was established by comparison of NMR, MS, and optical activity. Pure streptazolin was evaluated for its antimicrobial effects against ATCC pathogenic germs obtaining, as known, a moderate activity. However, recent studies have focused on the role of this compound as an antibiotic adjuvant. A sequential modelisation using PBD and CCD statistical methods allowed to maximize the antimicrobial activity under the following conditions: KCl (0.01%), K_2_HPO_4_ (0.1%), and MgSO_4_·7H_2_O (0.02%) with 9 days of incubation for inhibiting *Salmonella Typhi* ATCC 14028; KCl (0.051%) and MgSO_4_·7H_2_O (0.05%) with 5 days of incubation for improving effects against *Candida albicans* ATCC 10231.

Oligomycins A (**38**) and E (**39**) were produced as major metabolites by the *Streptomyces* sp. HG29 strain isolated from a Saharan soil collected in Hoggar (Tamanrasset, Southern Algeria) [104]. Their structure assignment is based on MS and NMR spectra, but no indication on the several stereocentres is given, neither are polarimetric data reported to allow a comparison with known oligomycins. Both metabolites have been already described to have a broad spectrum of bioactivies, mainly antifungal. Khebizi et al. [104] reported significant antifungal activity observed for **38** and **39** (with MIC values estimated between 2 and 75 µg/mL against representatives of the *Aspergillus*, *Fusarium* and *Penicillium* genera as well as *C. albicans*), but their known high toxicity to eukaryotic cells prevents any clinical applications.

A series of polyether antibiotics including nigericin (**40**), epinigericin (**41**), abierixin (**42**), and the new grisorixin methyl ester (**43**) were isolated from the *Streptomyces youssoufiensis* SF10 strain collected from Chélia Mountain, in Khenchela (North-eastern Algeria) [105]. The online coupled HPLC-ESIMS analysis provided the full polyether profile, and the preparative HPLC technique in the reversed phase condition gave pure compounds, which were identified by extensive NMR and ESI-MS spectra in comparison with reported data. Nigericin, the main member of the series, is known for its strong antibacterial antagonism and for its behavior as potassium ionophore, whereas the related metabolites grisorixin and abierixin exhibit weak activity against Gram-positive bacteria. A computational analysis on the structural epimerization at C-28 positon in the F ring of these metabolites (Figure 7) carried out by density functional theory (DFT) calculations allowed to compare their relative stability, providing structural considerations applicable to the other several members of the polyether class. The strain was cultured under different conditions (solid state or submerged fermentation, using several carbon sources, presence or absence of iron (II) sulfate, changing pH values, in co-culture with other *Streptomyces* species), and the production of nigericin present in the corresponding crude extracts was evaluated using a calibration curve by HPLC apparatus equipped with an evaporative light scattering detector (ELSD), a sensitive detector for the analysis of molecules lacking of chromophore units as nigericin. The best culture conditions provided a concentration of nigericin of 0.490 mg/mL in the extract. By the co-culture with *Streptomyces* sp., the formation of phenylacetic acid was observed, a metabolite previously reported from *S. humidus* cultures showing inhibition on some plant-pathogenic fungi. Otherwise, co-culturing SF10 strain with *S. coeruleorubidus* neither nigericin nor phenylacetic acid were observed [106].

Very recently, a novel *Saccharothrix xinjiangensis* ABH26 strain, isolated from a soil sample collected in the Adrar region (Southern Algeria), was studied by Lahoum et al. [107]. The new metabolites, named cyanogriside I (**44**) and J (**45**), were purified, and the suggested structures were indicated as a methoxy-bipyridine linked to sugar units through an O-oxime moiety. The long-range heterocorrelations by 2D-NMR experiments allowed to define the connectivities but not the configuration of the sugar unit. In the structures of cyanogrisides A–D, already reported from the marine-derived actinomycete *Actinoalloteichus cyanogriseus* WH1-2216-6, the sugar was fully assigned by X-ray diffractometric analysis [108] from nuclear Overhouser effect (NOE) experiments for cyanogrisides E–H [109]. Moreover, these last compounds showed different connectivities (the unit sugar replacing the methoxy group of **44**/**45**) so that cyanogriside names look like it is not strictly appropriate for metabolites **44** and **45**, and furthermore, their putative biosynthesis was not discussed in correlation with cyanogrisides A–H. Isolated from the same Algerian strain, Lahoum et al. reported also the identification of the known methoxy-bipyridines caerulomycin A (**46**), caerulomycin F (**47**), and caerulomycinonitrile (**48**). The five metabolites exhibited moderate inhibition on Gram-positive pathogenic bacteria and low effects on filamentous fungi and pathogenic yeast (with MICs > 100 µg/mL). Compound **47** emerged as the most active in the series, mainly on Gram-positive bacteria and fungi (MICs in the range 1–50 µg/mL corresponding to 4.6–230 µM).

Toumatia et al. [110] and, recently, Djinni et al. [111] isolated novel strains producing actinomycin D (**49**) and showed that genus *Streptomyces* isolated from Saharan soil of Ain amenas (*Streptomyces* sp. IA1) and Beni Abbes-Bechar (*Streptomyces* sp. GSBNT10) had potent antibacterial and antifungal activity against a wide range of plant pathogenic fungi. The production of the metabolite from *Streptomyces* sp. GSBNT10 was successfully enhanced using PBD and CCD methods, as detected by LC-MS analysis of crude extracts. Under the optimized culture conditions, a 58.56% increase of actinomicyn D formation was obtained, arriving at the value of 656.46 mg/L. These results suggested interest in scaling-up the process for access to this molecule, which is currently employed to treat some highly aggressive tumors, alone or in combined chemotherapies [111].

Messis et al. [112] used the Box–Behnken design approach to improve the antifungal activity of the *Streptomyces* sp. TKJ2 strain collected from a forest soil origin, but this study did not include the isolation and structural elucidation of the bioactive metabolites. 

The same modeling approach, including optimization of pH and temperature values, was applied to select the factors affecting antifungal activity of the *Streptomyces* sp. SY-BS5 strain isolated from an arid soil sample collected in Bou-Saada [51]. Similarly, the *Streptomyces albidoflavus* S19 strain, derived from wastewater collected in Bejaia region, was studied as antifungal producer. In detail, the best conditions for the production of anti-*Candida albicans* compounds were selected, evaluating a rise from 13 to 34 mm of the diameter inhibition zone. The data have highlighted the requirements of next studies to characterize the metabolites responsible for this activity [113].

In summary, the biological evaluation on the metabolites isolated so far from Algerian actinobacteria is mainly focused on the first bacterial and fungal inhibition, with the aim to find solutions to the urgent problems of increased bacterial resistance and of the incidence of fungal infection even potentially lethal in immuno-compromised people. Regarding antibacterial metabolites, studies are currently underway on their contribution in improving the efficacy of therapeutic antibiotics when used in combination with them. A few other reports are on their potential role as antitumor agents, also based on present studies of known antibiotics as promising agents able to inhibit in vitro and in vivo the development of human tumors. 

### 4.2. Other Activities 

#### 4.2.1. Tumor Cells Growth Inhibitors 

Actinobacteria are responsible for more than half of cytotoxic compounds of microbial origin approved in cancer therapy [113,114]. Few studies have focused on finding cytotoxic compounds derived from Algerian microorganisms and actinobacteria, except for cases of some cytotoxic antibiotics.

The polypeptide lactone actinomycin D (**49**), also known as dactinomycin, was the first antibiotic presenting anticancer activity and is now commonly used as a drug in mono and combined therapy in the treatment of a variety of highly aggressive malignancies, including Wilm’s tumor and Ewing’s sarcoma [115]. It is known for its inhibitory effect of cellular transcription by intercalating between adjacent base pairs in DNA. It was first identified from *Actinomyces antibioticus* in 1940s, later produced by various *Streptomyces* and *Micromonospora* species in the world, and also isolated from the Algerian desert soil (Ain Amenas and Beni Abbes)-derived *Streptomyces* strains *Streptomyces* sp. IA1 [110,111], demonstrating the effectiveness of the compound for biocontrol against chocolate spots of field bean and *Fusarium* wilt of flax diseases. 

Recently, the polyether antibiotics nigericin (**40**) and the new grisorixin methyl ester (**43**) isolated from *Streptomyces youssoufiensis* SF10 have provided significant cytotoxic activities against glioblastoma stem cells, with a higher activity for grisorixin methyl ester (GI_50_ values of 3.85 and 3.05 μM for two human glioblastoma stem cell lines), corresponding to a higher growth-inhibion cell-proliferation than the drug temozolomide [105]. The data are remarkable due to both the nature of glioblastoma multiforme as the most malignant primary brain tumors and the effect against stem cells which are resistant to the conventional therapies. Nigericin had also shown activity in suppressing colorectal cancer metastasis [116].

#### 4.2.2. Plant-Growth-Promoting Agents

Among actinobacteria-derived metabolites, the plant-growth-stimulating agents play important roles in agriculture, both in improving plant growth and in controlling or inhibiting phytopathogens infecting host plants. A number of reports on the isolation of plant-associated endophytic actinobacteria, mainly of *Streptomyces* genus [38] from various plants families and even soil [117], have been reported. It was described their metabolic potential as biological control agents and plant-growth promoters [118], which can replace chemicals and pesticides. In detail, according to Rugthaworn et al. [119], the biocontrol effect of actinobacteria can be either by lysis of fungal cell walls or by antibiosis through their capability of growth inhibition, competition, or hyperparasitism on several plant pathogenic fungi.

A plant-growth-promotion effect on seed germination and root elongation was observed by Goudjal et al. [38] through the production of indole-3-acetic acid (**50**), a phytohormone which is widespread among bacteria. This metabolite acts as a common natural auxin produced by the L-tryptophane metabolism pathway for eighteen strains of *Streptomyces* isolated from five spontaneous desert plants well adapted to the arid climatic conditions of the Algerian Sahara. The highest produced amount was estimated at 127 μg/mL by cultivating *Streptomyces* sp. PT2 strain in yeast extract-tryptone broth supplemented with 5 mg of L-tryptophane/mL. Moreover, Goudjal et al. [33] isolated two potent strains (CA-2 and AA-2 related to *Streptomyces mutabilis* NBRC 12800^T^ and *S. cyaneofuscatus* JCM 4364^T^, respectively) from native Algerian Saharan plants roots, which exhibited both in vivo biocontrol potential on *Rhizoctonia solani* damping-off, a largely common fungal pathogen affecting a wide range of crops seedlings, and the promotion of tomato plant growth. Similarly, Zamoum et al. [34] reported the production of indole-3-acetic acid and siderophores by the endophytic strain, *Streptomyces caeruleatus* ZL2. They observed the enhancement of tomato plant resistance to *Fusarium oxysporum* f. sp. *radicis lycopersici* root rot as well as the ability to promote seedlings growth, proposing therefore the possible application of the isolate ZL2 in crop protection. Furthermore, the study carried out by Toumatia et al. [120] on plant-growth-stimulating properties of the *Streptomyces mutabilis* IA1 strain derived from Saharan soil, demonstrated a potent and promising protective effect on wheat seedlings against *F. culmorum*, which is the causal agent of seedling blight, showing its growth-promoting ability by the production of indole-3-acetic acid and gibberellic acid (GA3). The study by Merrouche et al. [118] allowed to highlight the potent antifungal effect of *Saccharothrix algeriensis* NRRL B-24137 due to the production of dithiolopyrrolones compounds acting against *Fusarium oxysporum*, which induces wilt disease affecting flax, lentil, chickpea, and tomato.

A study by Goudjal et al. [33] on endophytic actinobacteria pertaining to *Streptomyces* genus, collected from spontaneous Saharan plants, allowed the isolation of indole-3-acetic acid (**50**) and showed a growth-promoting activity of tomato plants. 

## 5. Conclusions

In the last years, a growing interest in the exploration of less studied environments (such as marine, forest, sebkha, and arid ecosystems) and of the symbiotic associations has been observed in Algeria for isolation of new actinobacteria species and the isolated bioactive metabolites. This report provides the first comparative overview of the full diversity of actinobacteria phyla reported from the Algerian ecosystems. Compared to all geographical niches which provided 29 novel species, it is evident a high abundance of new actinobacteria species is associated with Algerian Saharan soil, yielding 27 novel species belonging to 15 genera. Fifty secondary metabolites have been isolated and identified, including 17 new molecular structures (**1**–**3**, **6**–**8**, **10**–**15**, **18**, **21**, **31**, and **43**–**45**), and then evaluated for their biological activities, mainly focusing on antibacterial and antifungal but also including cytotoxicity and promotion of plant growth. The following points are proposed and emphasized for future research in this topic: (i) the exploration of understudied ecological niches (telluric and marine) including associations of diverse nature as well as the reinforcement of the Algerian desert investigations; (ii) the investigation of the actinobacteria diversity in these ecosystems; (iii) the developement of more suitable cultivation techniques for the isolation of new and rare actinobacteria species from these niches; (iv) in-depth metabolic and genomic studies of new isolated species; and (v) the development of new biotechnologically exploitable species.

## Figures and Tables

**Table 1:** List of novelly discovered rare actinobacteria species isolated from different ecosystems in Algeria since 2002.

Microorganism	Ecological Niches and Climate	References
*Saccharothrix algeriensis* sp. nov.	Saharan Palm grove, Adrar; Saharan climate	[24]
*Mycobacterium algericum* sp. nov.	Goat-lung lesion, Souk El Tenine slaughterhouse, Bejaia; Mediterranean climate	[59]
*Actinopolyspora algeriensis* sp. nov.	Saharan salin soil, Ouargla; Saharan climate	[44]
*Actinopolyspora saharensis* sp. nov.	Saharan soil, El Oued; Saharan climate	[45]
*Actinopolyspora righensis* sp. nov.	Saharan soil, El Oued; Saharan climate	[46]
*Actinopolyspora mzabensis* sp. nov.	Saharan soil, Ghardaia; Saharan climate	[47]
*Saccharothrix saharensis* sp. nov.	Saharan palm grove, Adrar; Saharan climate	[60]
*Saccharothrix hoggarensis* sp. nov.	Saharan soil, Hoggar–Tamenrasset; Saharan climate	[61]
*Saccharopolyspora ghardaiensis* sp. nov.	Saharan soil, Ghardaia; Saharan climate	[48]
*Halopolyspora algeriensis* comb. nov.	Saharan soil, Mzab region, Ghardaia; Saharan climate	[62,63]
*Streptomonospora algeriensis* sp. nov.	Soil sample, Djelfa; semiarid climate	[49]
*Actinokineospora mzabensis* sp. nov.	Saharan soil, Beni izguen region, Ghardaia; Saharan climate	[64]
*Bounagaea algeriensis* gen. nov., sp. nov.	Saharan soil, El-Goléa, Ghardaia; Saharan climate	[50]
*Actinopolyspora biskrensis* sp. nov.	Saharan soil, Biskra; Saharan climate	[65]
*Prauserella isguenensis* sp. nov.	Saharan soil, Beni izguen region, Ghardaia; Saharan climate	[66]
*Nocardiopsis algeriensis* sp. nov.	Saharan soil, Adrar; Saharan climate	[67]
*Actinoalloteichus hoggarensis* sp. nov.	Saharan soil, Hoggar region, Tamanrasset; Saharan climate	[68]
*Saccharothrix tamanrassetensis* sp. nov.	Saharan soil, Hoggar region, Tamanrasset; Saharan climate	[69]
*Streptosporangium algeriense* sp. nov.	Saharan soil, palm grove in Adrar; Saharan climate	[70]
*Actinomadura algeriensis* sp. nov.	Saharan soil, Hoggar region, Tamanrasset; Saharan climate	[71]
*Mycobacterium icosiumassiliensis* sp. nov.	Water lake surface, Algiers; Mediterranean climate	[72]
*Actinomadura adrarensis* sp. nov.	Saharan soil, Adrar; Saharan climate	[73]
*Saccharothrix isguenensis* sp. nov.	Saharan soil, Mzab region, Ghardaia; Saharan climate	[74]
*Actinophytocola algeriensis* sp. nov.	Saharan soil, Mzab region, Ghardaia; Saharan climate	[75]
*Streptosporangium becharense* sp. nov.	Saharan soil, Beni Abbes region, Bechar; Saharan climate	[76]
*Streptosporangium saharense* sp. nov.	Saharan soil, Mzab region, Ghardaia; Saharan climate	[77]
*Saccharothrix ghardaiensis* sp. nov.	Saharan soil, Mzab region, Ghardaia; Saharan climate	[78]
*Planomonospora algeriensis* sp. nov.	Saharan soil, Beni Abbas, Bechar; Saharan climate	[79]
*Streptomyces massilialgeriensis* sp. nov.	Saline soil, dry lake, Oum el Bouaghi; semiarid climate	[80]

**Table 2:** Bioactivities of Actinobacteria metabolites derived from Algerian ecosystems.

Compound	Bioactivity	Producer Strain	Source	Reference
3-Methyl-2-butenoylpyrrothine (**1**), Tigloylpyrrothine (**2**), *n*-Butyropyrrothine (**3**), iso-Butyropyrrothine (**4**), Thiolutin (**5**)	antibacterial, antifungal	*Saccharothrix* sp. SA 233	Saharian palm grove soil (Adrar)	[24,81]
Valerylpyrrothine (**6**), Isovalerylpyrrothine (**7**), Formylpyrrothine (**8**), Aureothricin (**9**)	antibacterial and antifungal	*Saccharothrix algeriensis* NRRL B-24137, fully sequenced strain	Saharan soil	[82]
Crotonyl-pyrrothine (**10**), Sorbyl-pyrrothine (**11**), 2-Hexenyl-pyrrothine (**12**), 2-Methyl-3-pentenyl-pyrrothine (**13**)	antibacterial and antifungal	*Saccharothrix algeriensis* NRRL B-24137	Saharan soil	[83]
Benzoyl-pyrrothine dithiolopyrrolone (**14**)	antibacterial, antifungal	*Saccharothrix algeriensis* NRRL B-24137	palm grove soil (Southern Algeria)	[84]
Mutactimycin PR (**15**), Mutactimycin C (**16**)	moderate anti-Gram-positive bacteria	*Saccharothrix* sp. SA 103	Saharan soil sample (Tamanrasset, South Algeria)	[85]
ZA01 (**17**), ZA02 (**18**)	antibacterial antifungal	*Nocardiopsis* SA 103	non-rhizospheric soil samples, Saharan regions	[86]
Angucyclinone R2 (**19**)	antibacterial, antifungal, antitumor, antiviral, enzyme inhibitor, platelet aggregation inhibitor	*Streptosporangium* sp. Sg3	Saharan soil, Adrar region	[87,88,89]
D(-)-threo chloramphenicol (**20**)	antibacterial	*Saccharothrix* sp. PAL54	Saharan soil of Ghardaı¨a	[91]
2-Hydroxy-5-((6-hydroxy-4-oxo-4H-pyran-2-yl)methyl)-2-propylchroman-4-one (**21**), Phaeochromycins B (**22**), C (**23**), E(**24**)	antibacterial, anti-inflammatory	*Streptomyces sundarbansensis* WR1L1S8	Endophitic strain, inner tissue of marine algeae *Fucus* sp.	[53]
Compound A4 (**25**), Compound A5 (**26**)	anti-Gram-positive and -negative bacteria, anti-phytopathogenic and toxinogenic fungi	*Saccharothrix* SA198	Saharan soil, Tamanrasset (Southern Algeria)	[93]
Saquayamycin A (**27**), Saquayamycin C (**28**)	antifungal and antibacterial	*Streptomyces* spp. PAL114	Saharan soil, Béni-isguen-Ghardaïa (South of Algeria).	[94]
Vineomycin A1 (**29**), chaetoglobosin A (**30**)	antibacterial and antifungal	*Streptomyces* sp. PAL114.	Saharan soil	[95]
Novel hydroxamic acid (**31**)	antibacterial	*Streptomyces* WAB9	Saharan soil, Bechar	[96]
Di-(2-ethylhexyl) phthalate (**32**)	antibacterial	*Streptomyces* sp. G60	Saharan soil, Ghardaïa	[97]
2,4-Di-tert-butylphenol (**33**)	against *Candida* *albicans* and other pathogenic fungi	*Streptomyces mutabilis* G61	Soil sample Metlili, Ghardaïa	[99]
AT37-1 (**34**)	against multidrug-resistant *S. aureus*	*Streptomyces* sp. AT37	Saharan soil sample (Adrar)	[100]
(−)-7-Deoxy-8-*O*-methyltetrangomycin (**35**), (−)-8-Methyltetrangomycin (**36**)	anti-Gram-positive bacteria, antifungal	*Nocardiopsis* sp. HR-4	Salt-lake soil, Sebkha of Ain Salah (Saharan desert)	[103]
Streptazolin (**37**)	antimicrobial adjuvant antibiotic	*Streptomyces thermoviolaceus* SRC3	Fresh water river sediments	[43]
Oligomycin A (NK1) (**38**), Oligomycin E (NK2) (**39**)	anti-Gram-positive bacteria, antifungal	*Streptomyces* sp. HG29	Saharan soil sample (Hoggar, Tamanrasset)	[104]
Nigericin (**40**), Epinigericin (**41**), Abierixin (**42**), Grisorixin methyl ester (**43**)	glioblastoma stem-cell inhibitor	*Streptomyces youssoufiensis* SF10	soil derived	[105]
Cyanogriside I (**44**), Cyanogriside J (**45**), Caerulomycin A (**46**), Caerulomycin F (**47**), Caerulomycinonitrile (**48**)	anti-Gram-positive bacteria, antifungal	*Saccharothrix xinjiangensis* ABH26	Saharan soil (Adrar)	[107]
Actinomycin D (**49**)	antimicrobial, antitumor	*Streptomyces* sp. IA1. *Streptomyces* sp. GSBNT10	Saharan soil (Ain amenas) Saharan soil (Beni Abbes-Bechar)	[110,111]
Indole-3-acetic acid (**50**)	plant-growth-promoting activity	*Streptomyces* sp. PT2	Spontaneous herbaceous plants (*Cleome arabica*, *Solanum nigrum*, *Astragallus armatus*, *Aristida pungens*, and *Panicum turgidum*) (Sahara, Hassi R’mel region)	[38]
